# Success and Pitfalls of Genetic Testing in Undiagnosed Diseases: Whole Exome Sequencing and Beyond

**DOI:** 10.3390/genes14061241

**Published:** 2023-06-10

**Authors:** Valeria Barili, Enrico Ambrosini, Vera Uliana, Melissa Bellini, Giulia Vitetta, Davide Martorana, Ilenia Rita Cannizzaro, Antonietta Taiani, Erika De Sensi, Patrizia Caggiati, Sarah Hilton, Siddharth Banka, Antonio Percesepe

**Affiliations:** 1 Medical Genetics, Department of Medicine and Surgery, University of Parma, 43126 Parma, Italy; 2 Medical Genetics, University Hospital of Parma, 43126 Parma, Italy; 3 Department of Pediatrics and Neonatology, Guglielmo da Saliceto Hospital, 29121 Piacenza, Italy; 4 Medical Genetics, University of Bologna, 40138 Bologna, Italy; 5 Division of Evolution, Infection & Genomics, School of Biological Sciences, Faculty of Biology, Medicine & Health, The University of Manchester, Manchester M13 9PL, UK; 6 Manchester Centre for Genomic Medicine, Saint Mary’s Hospital, Manchester University Foundation NHS Trust, Health Innovation Manchester, Manchester M13 9WL, UK

**Keywords:** neurodevelopmental disorders, Cornelia de Lange syndrome, *MEIS2*, Kabuki syndrome, Kleefstra syndrome, episignature, chromatinopathies

## Abstract

Novel approaches to uncover the molecular etiology of neurodevelopmental disorders (NDD) are highly needed. Even using a powerful tool such as whole exome sequencing (WES), the diagnostic process may still prove long and arduous due to the high clinical and genetic heterogeneity of these conditions. The main strategies to improve the diagnostic rate are based on family segregation, re-evaluation of the clinical features by reverse-phenotyping, re-analysis of unsolved NGS-based cases and epigenetic functional studies. In this article, we described three selected cases from a cohort of patients with NDD in which trio WES was applied, in order to underline the typical challenges encountered during the diagnostic process: (1) an ultra-rare condition caused by a missense variant in *MEIS2*, identified through the updated Solve-RD re-analysis; (2) a patient with Noonan-like features in which the NGS analysis revealed a novel variant in *NIPBL* causing Cornelia de Lange syndrome; and (3) a case with de novo variants in genes involved in the chromatin-remodeling complex, for which the study of the epigenetic signature excluded a pathogenic role. In this perspective, we aimed to (i) provide an example of the relevance of the genetic re-analysis of all unsolved cases through network projects on rare diseases; (ii) point out the role and the uncertainties of the reverse phenotyping in the interpretation of the genetic results; and (iii) describe the use of methylation signatures in neurodevelopmental syndromes for the validation of the variants of uncertain significance.

## 1. Introduction

The use of the whole exome sequencing (WES) for the diagnosis of rare diseases is spreading worldwide and is becoming the most important diagnostic tool for understanding the molecular etiology of genetic conditions with developmental anomalies, including malformations and neurodevelopmental disorders (NDDs) [1]. This latter term includes psycho-motor impairments, intellectual disability, autism spectrum disorder, attention/hyperactivity defects and specific learning deficits [2]. NDDs are featured by a high clinical and genetic heterogeneity, which makes the identification of a genetic causative factor often challenging [1].

WES enables the detection of single nucleotide variants, insertions/deletions and copy number variants (CNVs) allowing a ~40% diagnosis, especially in those cases in which a trio/parental analysis is applied [3,4]. Trio strategy is fundamental for the interpretation of variants in NDD through information related to the familial segregation, by revealing the carrier state of the parents in recessive cases, both autosomal and X-linked, and by demonstrating the parental or de novo origin of heterozygous variants in syndromes with an autosomal dominant transmission pattern [5,6]. Moreover, in negative cases, several studies highlighted the relevance of a periodic re-analysis, which adds a further diagnostic yield up to ~20% [7,8,9,10,11,12].

Achieving a genetic diagnosis eases family planning, highlights the disease course, avoids unnecessary laboratory tests and helps in the interpretation of related medical conditions, such as seizures, hypotonia, facial dysmorphism and neuroimaging abnormalities [2]. Nonetheless, the diagnostic yield of WES did not show a substantial increase in the last decade of use of the technique, for instance, due to yet unknown disease genes or due to the difficulties in the interpretation, which may cast doubts on the pathogenicity of the DNA changes, which are defined as variants of uncertain significance (VUS). A periodic reanalysis of the role of the VUS is required, which will take into account the enrichment of the international databases and will benefit of improved pipelines of data filtration and analysis. In the attempt to enhance the WES diagnostic rate, four European Reference Networks (ERN-ITHACA, ERN-RND, ERN-Euro NMD, ERN-GENTURIS) built up the Solve-RD Project for the reanalysis of unsolved rare diseases [13,14] or collective variants [15]. Moreover, functional tests for the interpretation of the role of variants (mostly non truncating) in the aetiology of the disease are also strongly suggested, and they are also recommended by the ACMG–AMP (American College of Medical Genetics—Association for Molecular Pathology) guidelines [16,17,18]. One of the most recent examples of functional test was used for understanding the role of variants in genes encoding for chromatin-regulating proteins complex, causing a group of NDDs called chromatinopathies [19,20,21,22,23,24,25,26,27], which include Cornelia De Lange [28,29,30], Coffin–Siris [22] and Kabuki syndromes [28,31]. By the analysis of the genomic methylation status of the CpG at multiple loci across the genome, an “episignature” can be defined, thus demonstrating or excluding the presence of a derangement in the DNA methylation profile [22,23,24,25,26,27].

Here, we presented three clinical cases which showed the complexity of genetic testing in NDDs when using trio WES, and different approaches to increase the diagnostic yield including reverse phenotyping, reanalysis and episignature. Moreover, the reported patients may be helpful from a clinical point of view, since they refer to rare syndromes or atypical phenotypes of known syndromes.

## 2. Materials and Methods

### 2.1. Patient Inclusion

In compliance with the local ethical guidelines and the Declaration of Helsinki, all individuals provided informed consent for genetic analysis and results publication. Ethical review and approval were waived for this study because, according to the local policy, informed consent is considered sufficient for reports of an observational nature concerning a limited number of patients.

### 2.2. Whole Exome Sequencing

The genomic DNA was extracted from peripheral blood using Maxwell Blood DNA Purification System, quantified by Qubit dsDNA High Sensitivity (Invitrogen, Waltham, MA, USA).

Whole exome sequencing was performed starting from 50 ng genomic DNA with 37 Mb Nextera Rapid Capture Exome v1.2 (for cases #1 and #3) (Illumina, San Diego, CA, USA), for library preparation with enzymatic tagmentation, which uses a transposase to simultaneously fragment and insert adapters onto dsDNA, indexing PCR, clean-up, pooling, target enrichment with double-stranded DNA probes and post-capture PCR amplification/quality control following standardized protocols as per manufacturer guidelines.

For case #2, WES was executed starting from 50 ng genomic DNA with 36.5 Mb Twist Human Core 2.0 Exome Plus kit (Twist Bioscience, South San Francisco, CA, USA), for library preparation with enzymatic fragmentation/end-repair/dA-tailing, indexing PCR, clean-up, pooling, target enrichment with double-stranded DNA probes and post-capture PCR amplification/quality control according to the manufacture’s protocol. Both WES approaches targeted >98% of the coding elements and flanking ±20 intronic nucleotides.

Libraries were quantified by Qubit dsDNA High Sensitivity (Invitrogen, Waltham, MA, USA) and purity was determined with High Sensitivity D1000 ScreenTape (Agilent, Santa Clara, CA, USA). The BaseSpace genomic platform (Illumina, San Diego, CA, USA) and the Dragen Enrichment software (Illumina, San Diego, CA, USA) were employed for the calling and annotating variants. NGS results were aligned to the GRCh37 and GRCh38 human reference genome. A 70-fold median sequence coverage on target regions was obtained and a minimum depth coverage of 30 was kept for analysis, according to the guidelines of the American College of Medical Genetics and Genomics. Gene variants were visualized by the Integrative Genome Viewer (IGV, Broad Institute of MIT and Harvard, Cambridge, MA, USA) and coverage was assessed. The following exclusion criteria were applied on Variant Interpreter (Illumina, San Diego, CA, USA) and/or eVai Variant Interpreter (EnGenome, Pavia, Italy): 1. gnomAD annotated mean allele frequencies > 1% [32]; 2. “benign” or “likely benign” on either ClinVar [33] or ACMG/InterVar database; 3. Variants annotated by the software as “low severity” with a pathogenicity score ≤ 2, based on the ACMG/AMP criteria and predicting tools [34]. Variants matching OMIM-HPO phenotypes were chosen.

### 2.3. WES Re-Analysis with Solve-RD

Human phenotype ontology (HPO)-standardized clinical features and FASTQ files of WES were uploaded to the RD-Connect Genome-Phenome Analysis Platform (GPAP; https://platform.rd-connect.eu/, accessed on 25 February 2021) for re-analysis [13,35]. The analysis steps included genome-wide (i.e., not restricted to the enrichment targets) variant calling with GATK [36] to the GRCh37/Hg19 genome build. Gene variants related to NDD were annotated and prioritized with the following parameters: (i) coverage depth ≥ 20 and genotype quality ≥ 20; (ii) gnomAD [32] allele frequency < 1%; (iii) “VUS”(ACMG, class 3) or “Likely pathogenic” (ACMG, class 4) or “Pathogenic” (ACMG, class 5) classification; (iv) presence in ClinVar [33]. Prioritized variants were interpreted in the context of the phenotype according to the HPO inserted features in line with the family segregation. Variant validation through Sanger sequencing was performed [37].

### 2.4. CpG Methyl-Arrays

All the procedures were performed in collaboration with the EpiPro project in the NHS Genomic laboratory Hub at Manchester University (https://mft.nhs.uk/nwglh/test-information/rare-disease/epipro-project/). In brief, bisulphite conversion of genomic DNA was carried out using Zymo EZ DNA Methylation Kit as per manufacturer’s instructions. Methylation data were generated using Infinium Methylation EPIC bead Chip Kit and the associated Illumina protocol (https://emea.illumina.com/) was analyzed using clinically validated EpiSign v3 (Canada) algorithms, as previously described [22,38,39]. Confidence scores for episignature analysis including moderate and high profiles were based on prediction models such as MVP-score, Euclidean clustering and multidimensional scaling [27,40,41]. All data were clinically reviewed and interpreted by a certified molecular geneticist trained in EpiSign analysis.

## 3. Results

From a cohort of 14 patients affected by neurodevelopmental disorders who underwent to trio WES analysis, five cases received a genetic diagnosis (35.7%), whereas the remaining nine were submitted to the RD-Connect GPAP as part of the Solve-RD project (6 cases) or were internally re-analyzed applying different filters and improved database and reverse-phenotyping data: this process allowed the identification of causative variants in two more cases (14.3%).

In the present report, we described three patients from our cohort as illustrative cases of successes and pitfalls of the implementation of WES in NDD patients.

CASE 1

Patient #1 was a 10-year-old female, born from non-consanguineous healthy parents. In the sixth month of life, she underwent surgical correction of Fallot tetralogy and atrial septal defect; one year later, surgical intervention was performed for cleft palate. The overall growth was normal, while psychomotor development was delayed, with absent speech at 5 years of age and wide-based gait. Brain MRI showed only a mild ventricular dilatation. Clinical genetic evaluation raised the suspect of a syndromic condition, considering the presence of bifid uvula, short philtrum, tented upper lip, full lower vermilion, broad hallux and hypertrichosis. There was no family history of heart defects, cleft palate or intellectual disability (Table 1).

**Figure 1:**
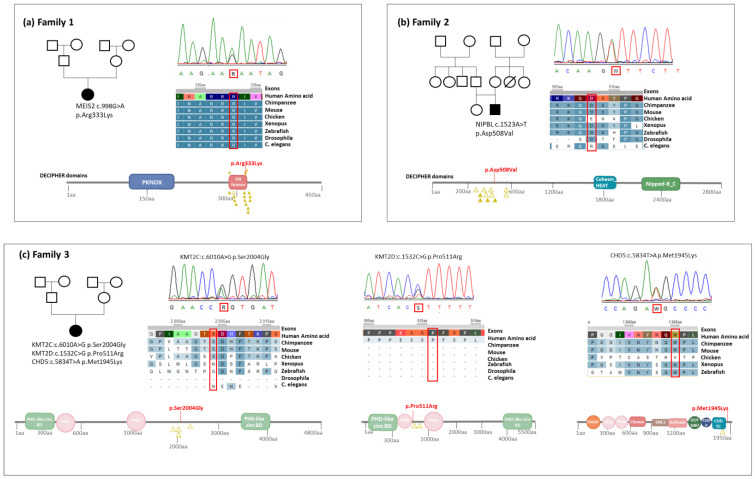
**Families and variants information.** (**a**–**c**) Family #1, #2 and #3 pedigrees, Sanger electropherograms for variants validation, amino acid sequence conservation between the different species and protein domains showing the proband’s variant and other missense changes described in Decipher and in ClinVar.

After negative results of the CGH array, WES on DNA extracted from the blood of the proband and the parents (trio) were performed, but the first attempt failed to identify a possible monogenic cause to the patient’s condition. Through the collaboration with the European Solve-RD project, we applied for a reanalysis of the case, which allowed the identification of the heterozygous c.998G>A (p.Arg333Lys) variant in the *MEIS2* gene, confirmed by Sanger sequencing (Figure 1a). The same variant was already described in association with a rare condition known with the initialism of CPCMR (cleft palate, cardiac defects and impaired intellectual development, OMIM #600987) [42] The variant was not found in both parents, defining a de novo origin (ACMG criteria were PS2, PM1, PM2, PM5, PP2 and PP3) and was scored as pathogenetic. Going back to the original analysis, we confirmed that the *MEIS2* variant was not present in the annotated report, suggesting an error in the bio-informatic pipeline or an issue related to the classification of the variant, considering that the first analysis was performed before the seminal article describing the CPCMR syndrome, at the end of 2018 [42].

CASE 2

Patient #2 was a 26-year-old male, born from non-consanguineous healthy parents, with a moderate intellectual disability. During the first years of life, he received a clinical diagnosis of Noonan syndrome, due to the co-presence of congenital heart defect (sub-aortic interventricular defect and interatrial defect), short stature and some suggestive dysmorphic features, such as short and slightly webbed neck, ptosis and micrognathia (Table 1). Karyotype and CGH-array were normal, a panel of genes related to RASopathies was tested and resulted negative. Although the overall gestalt was strongly suggestive of Noonan syndrome, the following clinical and instrumental evaluations revealed other features not typical of this condition, such as the presence of lacrimal ducts stenosis, bilateral renal dysplasia, small nipples, second and third toe syndactyly and hypoplasia of the corpus callosum. The morphology of the hands was atypical too, with hypoplasia of the distal phalanx of all the fingers and an abnormally low thumb placement.

We performed trio WES, which confirmed the absence of ACMG class 3-4-5 variants in genes related to Noonan syndrome or other RASopathies. However, by searching for de novo candidate variants, we identified the heterozygous c.1523A>T (p.Asp508Val) variant in *NIPBL* (Figure 1b), which was not found in both parents and in the general population database gnomAD. There were no reports of this variant in the literature or in public database, while a mutation involving an adjacent codon (p.Gln507His) was described as pathogenic in ClinVar. *NIPBL* has a low tolerance to missense variations, with a gnomAD constraint of missense upper Z-score for gene greater than 3.09. According to the ACMG classification, we reported this variant as Likely Pathogenic (ACMG criteria were PS2, PM2 and PP2). The previously mentioned clinical signs were highly compatible with the molecular diagnosis, with some distinctive features such as lacrimal ducts stenosis and small nipples, whereas the facial phenotype of the patient was not clearly suggestive of Cornelia del Lange syndrome (CDLS1), especially for eyebrows and body hair. Notably, pathognomonic CDLS1 features tend to become less evident when patients reach adulthood [43], and by analyzing the childhood pictures of the proband, we confirmed that his pediatric facial gestalt was more consistent with CDLS1. Finally, when we submitted the baby pictures to the Face2Gene algorithm (FDNA), the analysis suggested CDLS1 in the first four hypothesis, together with Noonan syndrome and other RASopathies. Many authors [44,45] described CDLS1 patients with atypical or milder phenotype as being associated with missense variants in *NIPBL*, corroborating the causative role of the p.Asp508Val missense variant in *NIPBL* for the patient’s condition.

CASE 3

Patient #3 was a 30-year-old female, born from non-consanguineous healthy parents, with a severe intellectual disability and plagiocephaly, surgically treated in the 4th month of life. Psychomotor and speech development was delayed, a brain MRI showed an asymmetry of the ventricular system and there was a dysmorphic appearance of the corpus callosum and cerebellar worm. After recurrent episodes of intestinal sub-occlusion, she was diagnosed with intestinal hypoganglionosis with immaturity of the ganglia. Other prominent features were delayed puberty (menarche at 18 years old), strabismus, dental crowding and several dysmorphisms, such as downslanting palpebral fissures, thick eyebrows, thin upper lip, thick lower lip, open mouth and brachydactyly of the hand and foot (Table 1). Height and cranial circumference were always between the 10th and the 25th pct, while weight was below the 3rd. Many genetic exams were performed during her childhood and young adulthood, starting from karyotype, CGH-array, sequencing of *FGFR2*, *FGFR3* and *TWIST1*, all of which were negative. Moreover, she was evaluated for Mowat–Wilson syndrome, but the analysis of *ZEB2* gene tested negative.

Suspecting a chromatinopathy, Trio WES did not identify any strong candidate pathogenic variants. However, two variants of uncertain significance were considered for further evaluation (Figure 1c): c.1532C>G (p.Pro511Arg) in *KMT2D* gene and c.6010A>G (p.Ser2004Gly) in *KMT2C*. Both were de novo variants, absent in general population and not reported in literature or public databases (ACMG criteria were PS2, PM2 and PP2 for *KMT2D* and PS2, PM2 and BP4 for *KMT2C*), and they were scored as variant of uncertain significance. To gain further elements for their clinical interpretation, an epigenetic analysis was performed, as explained in the Methods section and, more in detail, in Levy et al. [27]. The test resulted negative, showing no specific and known epigenetic signature: the two variants were then considered as probably neutral.

After a reanalysis, we identified another candidate de novo variant (Figure 1c), the c.5834T>A (p.Met1945Lys) in the *CHD5* gene (ACMG criteria were PS2, PM2, PP2 and PP3), encoding a chromodomain helicase DNA-binding protein, which was recently described in association with Parenti–Mignot neurodevelopmental syndrome [46]. The phenotype is partially overlapping, considering that three of the cases in the original study also had craniosynostosis, but the specific variant was never described in association with the condition. The variant was absent in the general population and the prediction tools lean towards a pathogenic significance, but the data regarding this gene are still too few and there is no specific episignature available at the moment.

## 4. Discussion

Currently, the molecular genomic characterization for diagnosis in NDDs is mainly based on the systematic use of WES, which achieves a previously unreachable diagnostic yield [1]. However, about half of the cases still remain unsolved mainly because of the unknown genetic basis of many diseases and the lack of functional validation assays for variants of uncertain significance. Moreover, the challenging phenotyping of patients with complex features, such as in NDDs, may impact on the proper selection of candidate genes, reducing the diagnostic yield of the test.

In the present study, we described three patients with NDD, to highlight the different challenges encountered in the diagnostic process. The first case (#1) showed the importance of the reanalysis: in fact, after a negative WES result in 2018, data were re-analyzed through the new platform developed by the Solve-RD project. This second attempt, after 3 years, led to the identification of the c.998G>A (p.Arg333Lys) variant in the *MEIS2* gene, causing the rare CPCMR (cleft palate, cardiac defects and impaired intellectual development) syndrome. Reported patients displayed overlapping facial characteristics (finely arched eyebrows, broad forehead, moderately shortened philtrum and tented upper lip) which, individually, are hardly recognized [47]. Interestingly, the proband’s missense variant impacts on a crucial amino acid in the *MEIS2 HOX* domain, which was reported as the only deleted residue (p.Arg333del) in a patient with a severe phenotype, comprising cleft palate and congenital heart defect, suggesting the importance of this amino acid for the protein’s DNA-binding capability [48].

It is known that a reanalysis of WES data after years using upgraded bioinformatic tools and databases increases the diagnostic yield in NDD by up to 20–30% after a negative first-tier analysis [49]. This higher rate is achieved owing to the continuous enrichment of database entries (such as ClinVar) and to the evolution of the analytical platforms, such as Solve-RD, which show the advantages of (1) using the human ontology-based phenotypes (HPO) [50], rather than the traditional diagnostic categories, (2) evaluating variant-specific rather than gene-specific phenotypes, with the potential to find functionally relevant mutations in addition to the classical, mainly disease-centered panel analysis.

In our cohort, a total of 7 out of 14 individuals (50%) received a confirmed genetic diagnosis: re-analysis allowed an improvement of 14.3% in final diagnosis, though numbers were too small to extrapolate significant statistical information. Our cases were analyzed after 1 to 3 years, although it was difficult to define a precise timeframe for reanalysis, since this need had to be commensurate with the human and technological resources of each laboratory: in this regard, studies centered on the automatization of the re-evaluation process are needed. Moreover, Case #1 also showed the role of reverse phenotyping, since it would have been difficult to clinically suspect CPCMR, an ultra-rare syndrome without the genetic data, even though a close phenotypic overlap exists among our patient and those reported in the literature. Reverse phenotyping also led to unexpected conclusions, such as in Case #2, who lived the first 25 years of his life with a clinical diagnosis of Noonan syndrome (NS), without a confirmation of the genetic testing results. Although this occurrence is not uncommon, since almost one out of four patients with Noonan-like features remains undiagnosed [51,52], our patient presented some specific clinical signs (lacrimal ducts stenosis, small nipples, hand conformation) which were not typical of NS but were used to switch the diagnosis towards CdLS after the finding of a rare, novel de novo variant in *NIPBL*. CdLS (OMIM # 122470, #300590, #610759, #614701 and #300882) is a multisystem NDD featured by craniofacial appearance, pre- and post-natal growth inhibition, intellectual and developmental deficit, behavioral issues and limb defects, all with a typically variable expressivity [53]. The diagnosis of CdLS was not straightforward because some of the most recognizable features of the syndrome, such as the synophris, were absent, as often happens in adult patients especially when missense variants occur [43,44]. In Case #2, reverse phenotyping, together with the retrieval of old family pictures, played a central role for reaching the correct diagnosis, highlighting the differences in the morphological features between the adult and the pediatric age in the same individual [54].

Finally, the clinical features of Case #3 showed similarities with those of the chromatinopathies, including developmental delay, facial dysmorphisms, intellectual disability and behavioral perturbation. Moreover, WES results suggested candidate variants in genes related to histone methylation, such as *KMT2D, KMT2C* and to the ATP-dependent chromatin remodeling complex, such as *CHD5*. Remarkably, genetic variants in the histone methyltransferase *KMT2D* were associated with Kabuki syndrome type 1, characterized by typical facial features, postnatal growth deficiency, organ malformations and NDD, whereas pathogenic variants in *KMT2C* were associated with Kleefstra syndrome (OMIM # 617768), an autosomal dominant chromatinopathy characterized by delayed psychomotor development, variable intellectual disability and mild dysmorphic features such as microcephaly, flattened midface and prominent eyebrows [55,56,57]. For both genes, the epigenetic analysis was negative, showing no specific and known signature: the two variants were then considered as probably neutral.

Retrospectively, the phenotype of our patient was not entirely compatible with those conditions, since features such as craniosynostosis and intestinal hypoganglionosis are not typical of Kabuki or Kleefstra syndrome. Moreover, these two syndromes usually display distinctive cranio-facial dysmorphisms, absent in our case [58,59]. Other compatible syndromes, such as Mowat–Wilson syndrome, were already ruled out by previous tests.

At the end, Case #3 remained unsolved, but the diagnostic process led to the exclusion of two candidate variants thanks to epigenetic studies and revealed a possibly causative variant in the chromodomain-helicase-DNA-binding protein 5 gene (*CHD5*). This gene was only recently described in association with an autosomal dominant NDD, which is characterized by impaired intellectual development, speech delay, motor delay and behavioral problems. Among the other features, craniosynostosis, a feature of our Case #3, was reported in some patients with *CHD5* variants, whereas epilepsy, absent in our patient, was described in more than 60% of the reported cases [46], not allowing a final pathogenic imputation for the *CDH5* variant in our patient and a phenotypic expansion of the syndrome. 

In conclusion, the thread among the reported cases resides in the difficult path towards a genetic diagnosis through WES analysis, highlighting the concept that negative results must be periodically challenged by their reanalysis, functional studies and by all the diagnostic tools which become available over time.

## Figures and Tables

**Table 1:** Summary of the clinical and genetic characteristics of the three patients.

	Patient F1	Patient F2	Patient F3
**Sex**	Female	Male	Female
**Age (years)**	10	26	30
**Candidate variants**	***MEIS2***(NM_170675.5):c.998G>A (p.Arg333Lys); P; absent; absent	***NIPBL***(NM_133433.3): c.1523A>T p.(Asp508Val); VUS; absent; absent	***KMT2C***(NM_170606.):c.6010A>G (p.Ser2004Gly); VUS; 0.00000398; absent.
(RefSeq, Protein, ACMG class, GnomAD frequency, ClinVar)			***KMT2D***(NM_003482.):c.1532C>G (p.Pro511Arg); VUS; 0.0000161; absent
			***CHD5***(NM_015557.3):c.5834T>A (p.Met1945Lys); LP; 0.00000973; absent
**Trasmission**	AD (De Novo)	AD (De Novo)	AD (De Novo)
**Phenotype:**			
Intellectual disability	Moderate/severe	Moderate	Moderate/Severe
Brain anomalies	+	+	+
Craniosynostosis	-	-	+
Facial dysmorphism	+	+	+
Cleft palate	+	-	-
Poor growth	-	+	-
Feeding difficulties	-	+	-
Cardiac defects	+	+	-
Renal defects	-	+	-
Limbs defects	-	+	+
Intestinal defects	-	+	+
**Final diagnosis**	CPCMR syndrome	Cornelia de Lange syndrome, type 1	Undefined chromatinopathy?
**Key features for clinical diagnosis**	Cleft palate, cardiac defect, impaired intellectual development	Lacrimal ducts stenosis, small nipples, hands conformation	NDD, thick eyebrows, thick lower lip, open mouth, hands conformation
**Challenges encountered**	Negative result at first WES trio analysis	Noonan-like features (short, webbed neck, ptosis, heart defect).	No pathogenic variants, but 3 VUS in chromatine-related genes
		Face changes (aging)	
**Key strategies adopted**	Reanalysis.	Reverse phenotyping.	Episignature.
	Reverse phenotyping	Face2Gene	Reanalysis

## Data Availability

All relevant data are available from the corresponding author upon request.
